# A fast algorithm to find reduced hyperplane unit cells and solve *N*-dimensional Bézout’s identities

**DOI:** 10.1107/S2053273321006835

**Published:** 2021-08-13

**Authors:** Cyril Cayron

**Affiliations:** a Laboratory of Thermo Mechanical Metallurgy (LMTM), PX Group Chair, EPFL, Rue de la Maladière 71b, Neuchâtel, 2000, Switzerland

**Keywords:** *N*-dimensional Bézout’s identity, hyperplane unit cell, integer relation, twinning

## Abstract

Deformation twinning on a plane is a simple shear that transforms a unit cell attached to the plane into another unit cell equivalent by mirror symmetry or 180° rotation. Thus, crystallographic models of twinning require the determination of the short unit cells attached to the planes, or hyperplanes for dimensions higher than 3. Here, a method is presented to find them. Equivalently, it gives the solutions of the *N*-dimensional Bézout’s identity associated with the Miller indices of the hyperplane.

## Abstract 2

The paper describes a method to determine a short unit cell attached to any hyperplane given by its integer vector **p**. Equivalently, it gives all the solutions of the *N*-dimensional Bézout’s identity associated with the coordinates of **p**.

## 1. Introduction   

**Figure 1:**
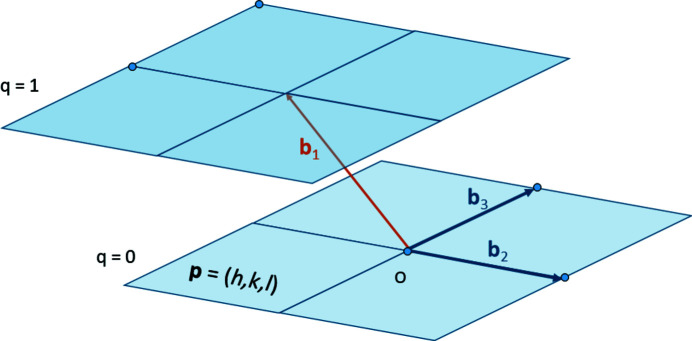
Unit cell associated with the plane 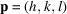. The out-of-plane vector 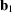 points to a node of the layer *q* = 1, and the in-plane vectors 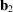 and 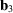 lie in the layer *q* = 0. The vector 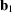 is a solution of the Bézout’s identity 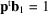, and the vectors 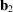 and 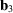 are solutions of the integer relations 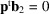 and 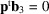.

How to determine a unit cell attached to a plane 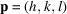
? This problem occurs for example in the crystallographic models of twinning, when the obliquity or the shear values must be calculated for many planes. It is intuitively solved for low-index planes, but the solutions are more difficult to obtain for high-index planes. In addition, if a unit cell can be found, can it be reduced to a smaller one? In dimension *N*, the difficulty of finding a small unit cell attached to a hyperplane of dimension 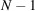 becomes even more pronounced. Let us express mathematically this ‘hyperplane unit-cell problem’ by the notations detailed in Appendix *A* [App appa]. We assume that a hyperplane is known only by its Miller indices 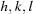 which are coprime integers, or equivalently by its normal which is expressed as an integer vector of coordinates 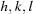 in the reciprocal space. We want to determine a small unit cell such that one short integer vector of the cell points to a node of the first layer parallel to the hyperplane, and the other 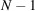 short integer vectors lie in the hyperplane. The ‘out-of-plane’ vector and the ‘in-plane’ vectors are noted 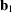 and 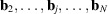, respectively. The vector 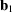 is such that 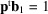, and the 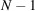 vectors 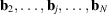 are such that 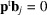. The coordinates of the vector 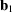 constitute a solution of the *N*-dimensional Bézout’s identity formed on the coordinates of 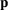. The coordinates of any of the vectors 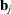, 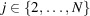, are solutions of what is called an ‘integer relation’ with the coordinates of 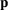 (Appendix *A* [App appa]). For example, with 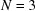, the integer coordinates 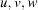 of 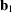 verify the equation 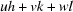 = 1; the integer coordinates 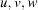 of the vector 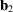 (or 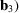 verify the equation 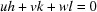, as illustrated in Fig. 1 ▸.

Finding solutions to integer relations is not complicated. For 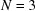, if we know a plane 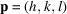 with let us say 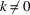, it is not difficult to find two integer vectors 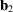 and 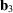 in this plane, for example, 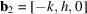 and 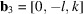. The difficult part of the problem is to find vectors with small coordinates by considering all the possible linear combinations of the Miller indices 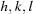. Finding the shortest solutions in dimension *N* is an NP-hard (non-deterministic polynomial-time) problem well known in computer science and cryptography. An algorithm called PSLQ gives short solutions to integer relations with any vector 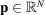 (Ferguson *et al.*, 1999 ▸; see also Wikipedia, 2021*a* ▸). It has permitted the discovery of numerous previously unknown identities among real numbers; one of them is the formula that allows the calculation of the *n*th hexadecimal digit of π without computing the preceding digits (Bailey *et al.*, 1997 ▸; Raayoni *et al.*, 2021 ▸). The algorithm presented in the present paper gives only solutions for the vectors 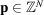 but, as we will show, the vectors we obtain are shorter than those obtained by PSLQ. Our algorithm actually provides simultaneously a short solution to the *N*-dimensional Bézout’s identity and short solutions to the ‘integer relations’. It gives the affine space of all the solutions of the *N*-dimensional Bézout’s identity. From a crystallographic point of view, it provides a small unit cell attached to a hyperplane.

Recently, Gorfman (2020 ▸) proposed a method to find some solutions to an intermediate problem that we will call the ‘column-constrained unimodular matrix’ (CCUM) problem in order to differentiate it from the initial ‘hyperplane unit-cell problem’. The CCUM problem consists of finding a uni­modular matrix **M** such that the first column is equal to a fixed integer vector **t**. We recall that a unimodular matrix has integer entries and its determinant is 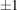. Note that, in Gorfman’s paper, it was the last vector (and not the first one) that was imposed, but that does not change the problem. Gorfman’s approach involves a series of multiplication with matrices called **S** containing 0, 1 and −1 in order to reduce the imposed vector **t** to a unit vector (a vector for which one of its coordinates is 1 and the others are 0). Gorfman showed that the same algorithm applied in the reciprocal space to a vector **p** gives a solution to the hyperplane unit-cell problem. Let us explain how it works with our notations. For an imposed reciprocal vector **p**, Gorfman’s method permits one to obtain a unimodular matrix 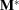 that has **p** for the first column vector. Then, the inverse of its transpose 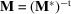 is calculated. Since 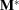 is a unimodular matrix, the matrix 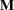 is also uni­modular, which implies that its columns are integer vectors. Let us call them 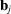. Since 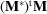 is the identity matrix, its first column is a vector that has 1 as the first coordinate and 0 for all the other coordinates. This means that 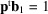 and 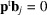 for 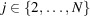, which proves that the matrix 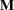 is a solution of the hyperplane unit-cell problem. Gorfman’s idea of using unimodular matrices is very interesting and his approach is innovative and inspiring, but it does not give short solutions. For example, for the plane 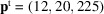, the solution determined by his algorithm in which the first imposed column vector is 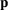 is 
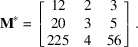
The inverse of its transpose is 
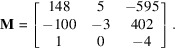
The reader can check that the scalar product of 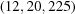 with the first column vector 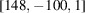 is 1, and that the scalar product with the last column vectors 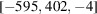 and 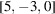 is 0. However, the vector 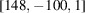 solution of the 3D Bézout identity and the vector 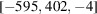 solution of the integer relation are large. The vector 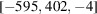 is even larger than the obvious solution 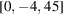. More generally, Gorfman suggests that the algorithm could be ‘an alternative approach to calculate the Bezout coefficients’, but we would like to show that the opposite approach is possible. The aim of the paper is to show that determination of the Bézout’s coefficients is an efficient way to find short solutions of both the CCUM problem and hyperplane unit-cell problem. The algorithm proposed in the present paper is based on Euclidean division. An algorithm to determine some short solutions to the *N*-dimensional Bézout’s identity is proposed in Section 2. The algorithm to solve the CCUM problem is detailed in Section 3. Sections 2 and 3 are independent. In Section 4, we explain how to combine the two algorithms to find short solutions to the hyperplane unit-cell problem. Some examples will be given and compared with the PSLQ algorithm. The method has been encoded in a Python 3.8 computer program called *GeneralizedBezout*. The examples given were obtained on a laptop computer equipped with an Intel Core i7-4600 CPU 2.1 GHz, 64-bit Windows system with a RAM of 8 GB. Note: the Python program *GeneralizedBezout* is freely available from the author upon request.

## 2. *N*-dimensional Bézout’s identity   

Given a set of integers 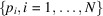 we look for another set of integers 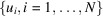 such that 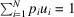. In other words, given an integer vector **p** of coordinates 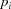, we want to get the coordinates 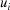 of an integer vector 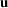 that is such that 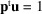. If *N* = 2, the fast and well known algorithm based on Euclidean division gives a solution that is also the shortest one (Capparelli, 2020 ▸; Wikipedia, 2021*b* ▸). Surprisingly, we could not find in the literature algorithms in high dimensions *N*. We propose here two recursive algorithms. They give different solutions that are all valuable, but we will see that the second one gives shorter solutions.

*Method-0*. We consider 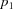 and 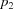 the two first coordinates of 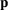, and we call 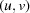 their Bézout numbers, *i.e.* 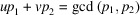. If we note 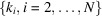 the Bézout numbers in dimension 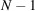 associated with the set 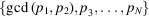, a solution of the *N*-dimensional Bézout’s identity is thus 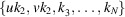. This method is easy to compute by recursion until the dimension decreases to *N* = 2 for which the solution is given by the classical Bézout’s algorithm. The problem related to this method is that the absolute values of the Bézout numbers 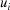 can be quite high. One could screen all the pairs 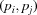 in place of 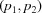 to determine the lowest Bézout numbers but this method would be unrealistic for high dimensions *N*. We could find another method for which the values are lower than those determined by method-0.

**Figure 2:**
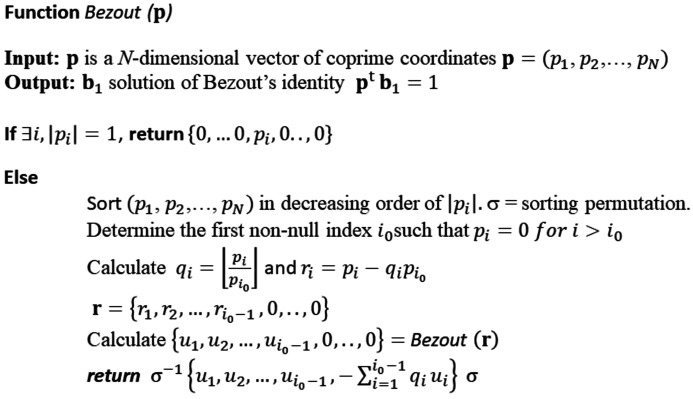
Pseudocode to find Bézout numbers associated with the coordinates of a vector **p**.

*Method-1*. We consider the set of integers 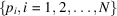. If 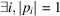, the solution of the Bézout identity is immediately 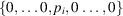. If none of the 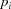
’s has 1 as absolute value, the set 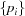 is sorted in decreasing order of the absolute values of 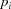. The sorting permutation σ is kept in memory. The smaller non-null value is called 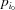. We calculate the quotient set and the residue set {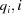 = 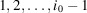} and 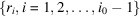 with 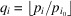 and 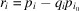, quotient and remainder of the Euclidean division by 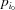. If we note 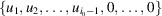 the Bézout numbers associated with the set {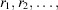 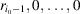}, a solution of the *N*-dimensional Bézout’s identity is 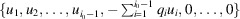. This method is easy to compute by recursion until one of the absolute values of the input vector is 1. The correct order of the Bézout numbers associated with the initial set 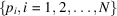 is restored by applying 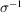. The pseudocode is given in Fig. 2 ▸.

The Bézout numbers calculated with method-1 are smaller than those obtained by method-0. Only method-1 will be considered in the rest of the paper. With the vector 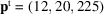, it gives 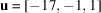. With the vector 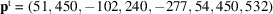, it gives 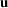 = [−3, 0, 0, 0, 0, −3, 0, 1]. The calculation lasts only a few ms. Even if method-1 gives small Bézout vectors **u**, it may not give systematically the smallest ones. We will see in Section 4 how ‘hyperplane shearing’ can give shorter Bézout vectors **u** with the help of the CCUM algorithm detailed in Section 3.

## 3. Algorithm to solve the column-constrained unimodular matrix problem   

### 3.1. Case where one of the coordinates of **t** is ±1   

Now we consider the CCUM problem. There is a simple and immediate solution if the first coordinate of 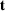 is 1. In that case, any diagonal or even triangular matrix **M** with 1 in the diagonal and with **t** as the first column checks the condition det(**M**) = 1. If the first coordinate of 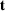 is −1, changing one 1 into −1 in the diagonal is sufficient to maintain det(**M**) = 1. The example used by Gorfman (2020 ▸) with the vector 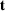 of coordinates 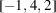 enters in this category. A direct solution is
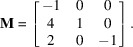
Note that the result is obtained without any calculation. If one of the coordinates of 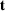 is 1 in a position *i* > 1, then a simple matrix of permutation **P** is sufficient to recalculate the matrix **M**. We will not give more details here because the solutions are actually included in the more general method based on Bézout’s identity explained as follows.

### 3.2. Case where **t** has at least one pair of coprime coordinates   

In the case *N* = 2, the general solution to the CCUM problem is given by the classical 2D Bézout’s identity. We note
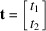
the imposed vector. There is a solution if and only if the integers 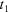 and 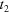 are coprime, and the solution is simply 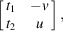
where *u, v* are the Bézout numbers associated with 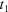 and 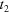, *i.e.* solutions of the equation 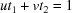. If 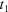 and 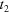 are not coprime, the determinant of any matrix **M** with **t** in the first column would be a multiple of gcd(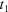, 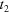), the greatest common divisor of 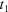 and 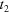, and thus cannot be equal to 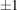. The resulting vector 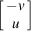
is the shortest vector. The other vectors are 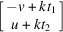
with 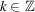.

Now, we consider the case where *N* > 2 and the vector **t** has its two first coordinates 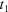 and 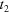 that are coprime numbers. We consider the matrix **M** made of two blocks, the top left one is 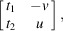
where *u, v* are the Bézout numbers associated with 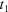 and 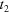, and the bottom right one is the 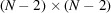 identity matrix. Then, the first column of **M** is replaced by **t** (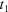 and 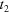 are not changed, and the zeros in **M**_*i*,1_ are replaced by 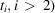. The matrix **M** is the solution of the CCUM problem.

When the two coprime coordinates of vector **t**, 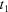 and 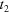, are not the first ones, the permutation matrices 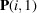 and 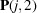 are used to return to the previous case. We recall that a permutation matrix 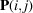 is a 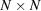 identity matrix, except for the line *i* for which 1 is written in the column *j*, and for the column *j* where 1 is written in the line *i*. Permutation matrices are unimodular matrices and are equal to their inverse. The unimodular matrix 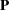 = 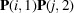 is such that the vector 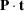 has for first coordinates the coprime numbers 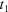 and 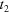. We thus return to the previous case. If we call **M** the two-block solution of that case, the solution of the problem is given by the matrix 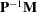. Note that 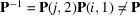.

With 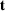 of coordinates 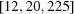, the algorithm gives 
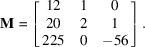
The algorithm works very efficiently, even in high dimensions and with large coordinates. For example, with 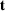 of coordinates [1551, −540, 67, −102, 2140, −277, 32, 366, 450, 1532], the algorithm gives immediately (less than 1 ms) a solution:
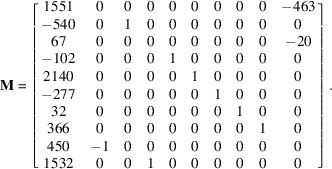



### 3.3. Case where none of the pairs of coordinates of **t** are coprime   

Now, let us consider the rarer cases in which none of the pairs 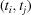 of coordinates of 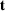 are coprime despite the fact that the set of coordinates of **t** are coprime (as mentioned previously, if they are not, there is no solution to the problem). The set of integers 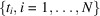 is said to be ‘coprime but not pairwise coprime’. A classical example is {6, 10, 15}. Let us recall that in large dimensions *N*, the probability that a set of integers 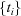 is coprime but not pairwise coprime is very small because the probability that two randomly chosen integers are coprime is quite high: it is equal to 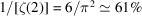, where 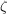 refers to the Riemann zeta function (Wikipedia, 2021*c* ▸). The exact calculation of the probability for a set of *N* integers 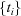 to be coprime but not pairwise coprime as a function of *N* is not straightforward and is beyond the scope of the present study. Even if rare, these cases can be solved as follows. We consider the two first coordinates 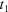 and 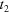 of the vector 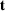 (any pair of coordinates would also work). As 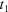 and 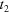 are not coprime, they can be written 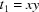 and 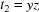, where *x, y, z* are three integers and 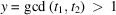. It is important to note here that there is at least another coordinate 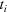 with 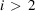 that cannot be divided by *y* because if it were not so the set 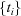 would not be coprime. We call 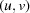 the Bézout numbers associated with 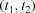, 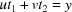. The pair 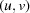 are also the Bézout numbers associated with 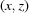, 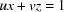, *i.e.* 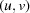 are also coprime. We call 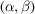 the Bézout numbers associated with 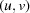. We consider the matrix 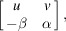
its determinant is 1, and 
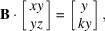
with 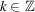. We build the 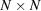 matrix 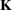 from the 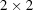 block 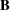 and from the 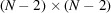 identity matrix. The first coordinate 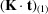 of the new vector 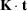 is coprime with at least one of the coordinates 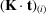 with 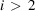. It means that the method described in Section 3.2 can be applied to calculate a matrix **L** such that 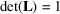, and its last column is the vector 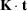. The matrix 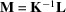 is then such that its determinant is also 1 and its last column is 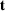. As the determinant of 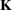 is 1, 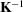 is the adjugate (transpose of the cofactor matrix) of **K**, and is thus an integer matrix. Consequently, 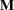 is also an integer matrix, solution of the problem.

**Figure 3:**
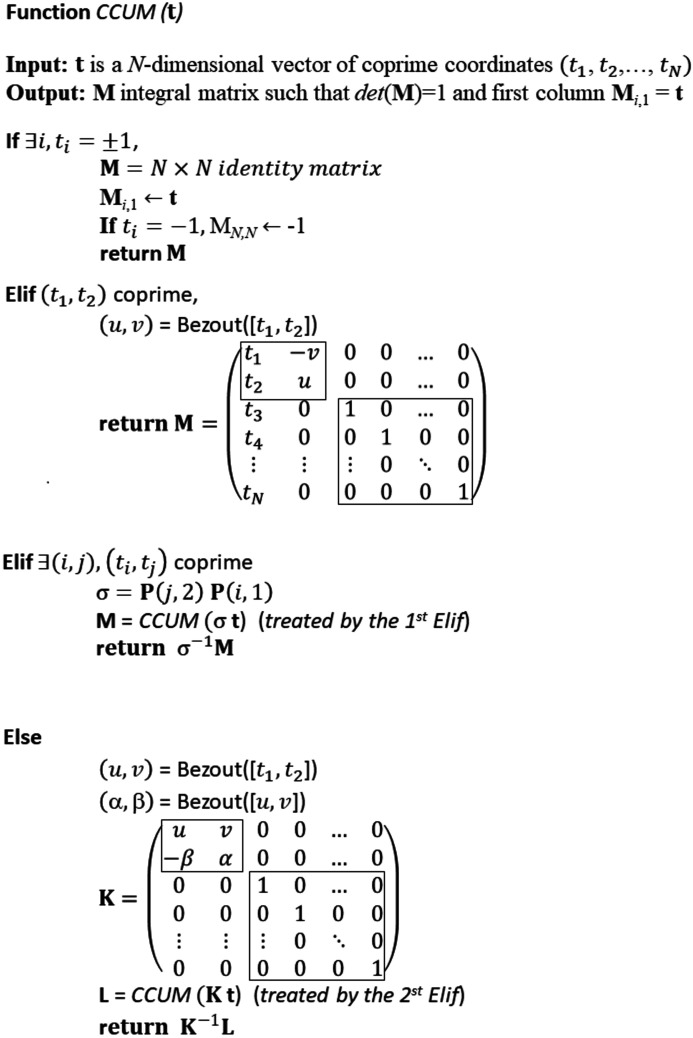
Pseudocode to find a column-constrained unimodular matrix associated with an integer vector **t**.

The algorithm is effective and fast, whatever the dimension *N* of the vector 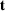. The pseudocode is shown in Fig. 3 ▸.

We give an example with the classical set of coprime but not coprime coordinates [6, 10, 15]. The algorithm gives immediately a solution (the vectors are written in columns):
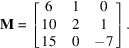



Let us build another example with a vector 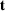 of coordinates 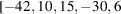]. A solution is
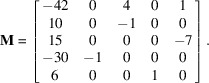



## 4. Hyperplane unit cell by oblique projection   

Let us recall the hyperplane unit-cell problem. We are looking for a set of *N* vectors 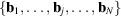 such that the first out-of-plane vector 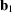 is such that 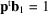 (pointing to a node of the layer 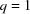), and the 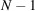 in-plane vectors 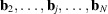 are such that 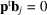 (lying in the layer 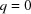). The solution 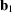 of Bezout’s identity is placed in the first position to be coherent with the notations we used in a separate paper dedicated to the lattice reduction (Cayron, 2021 ▸). The method we propose uses a short solution to the *N*-dimensional Bézout identity (Section 2) and a solution of the CCUM problem (Section 3).

**Figure 4:**
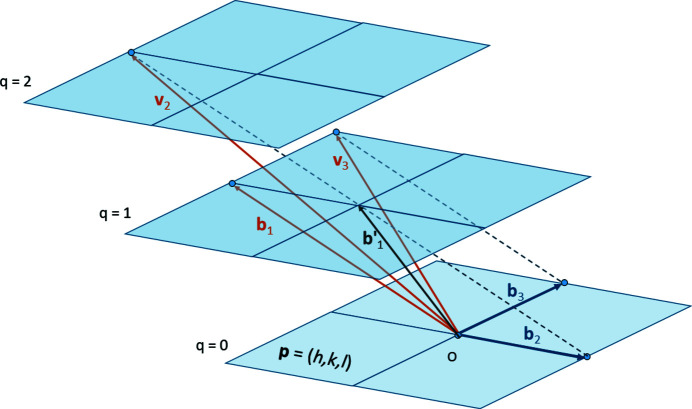
Projections of the vectors 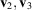 along 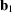 onto the plane 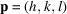. The vector 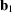 is a short solution of the Bézout’s identity 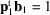. The vectors 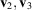 are the solutions of the 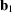
–CCUM problem. The vector 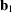 is a short vector that can be further shortened into a vector 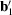 by ‘hyperplane shearing’ (Cayron, 2021 ▸).

We start from the input vector 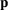. The coordinates of the vector 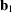 pointing to a node of the layer 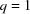 are the solution of the Bézout’s identity associated with 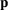. They are obtained by the algorithm detailed in Section 2 (method-1). Now, how to determine the 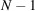 vectors in the layer 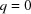
? We consider the unimodular matrix **M** that is such that the first column is the vector 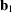. The 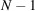 next column vectors of the matrix **M** are called 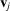 for 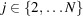. Each of these vectors belongs to the lattice; thus, they verify 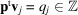. The vectors 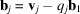 verify 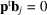 for 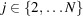; *i.e.* they are in-plane vectors lying in the layer *q* = 0. Geometrically, the vectors 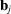 are obtained by oblique projection of the vectors 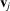 along 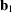 onto the plane 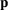, as illustrated for 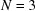 in Fig. 4 ▸.

**Figure 5:**
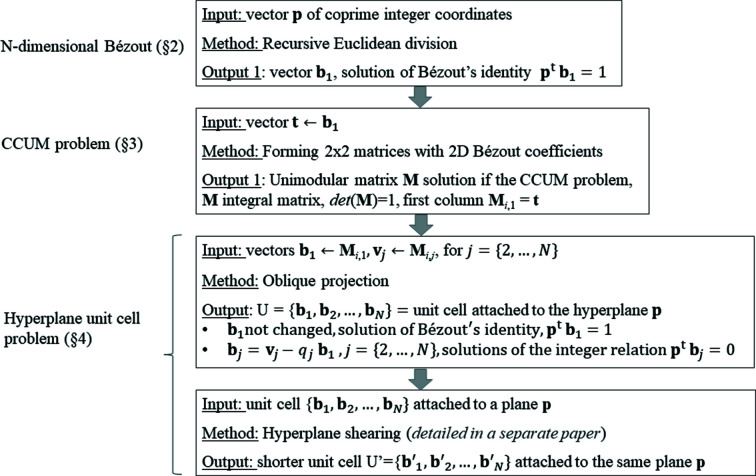
Pseudocode of the sequence of operations to determine a short unit cell associated with a hyperplane **p**. The unit cell is made of *N* short vectors 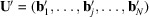, such that 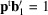 and 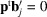.

Now, we have a cell 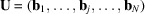 attached to the plane 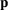 such that 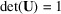, 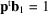 and 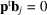 for 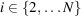. It is thus the unit cell we were looking for. As the vectors used for the projection are short, the unit cell is not large. It can be reduced even more. There are different methods to find a reduced unit cell 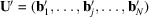, with 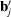 that have the same properties as **b**_*j*_ with the vector 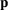, but with shorter lengths and with angles between them closer to orthogonality. One could apply for example the LLL algorithm well known in computer science (Lenstra *et al.*, 1982 ▸). We realized however that the algorithm developed in Section 4 can also be used to define the operation of ‘hyperplane shearing’ which consists of shearing the unit cell such that the vector 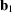 becomes 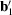 nearly normal to the plane 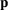 as illustrated in Fig. 4 ▸, and that this operation can be coupled with other lattice reduction methods to rival LLL implemented in *Mathematica*. The hyperplane reduction and its application to lattice reduction are detailed in a separate paper (Cayron, 2021 ▸). The pseudocode of the set of operations Bézout–CCUM–Projection–Hyperplane reduction is shown in Fig. 5 ▸.

The program written in Python 3.8 called *GeneralizedBezout* incorporates the lattice reduction operation described by Cayron (2021 ▸). Let us give some examples we obtained:

(i) With 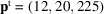. The Bézout vector associated with the plane 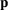 given by method-1 described in Section 2 is 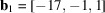. After determining a first unit cell by projections along 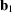, and after lattice reduction, this vector becomes 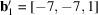. The final reduced unit cell is given by the matrix 
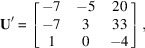
where the vectors are written in columns. The first vector is the short solution of the 3D Bézout’s identity and the other vectors are short solutions of the integer relations with the coordinates of **p**.

(ii) With 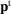 = (−54, 131, −48, 632, 23, 177, 333, 99, −581, 377). The coordinates were randomly chosen. The Bézout vector associated with the plane 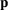 given by method-1 described in Section 2 is 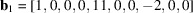. After determining a first unit cell by projections parallel to 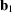, and after reducing this unit cell, this vector becomes 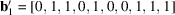. The reduced unit cell is
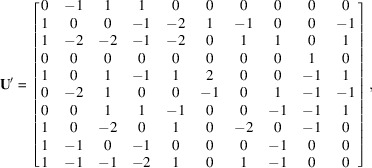
where the vectors are written in columns. The first vector is a short solution of the 10D Bézout’s identity and the other vectors are short solutions to the integer relations with the coordinates of **p**. The calculation lasted 20 ms. The PSLQ method implemented in *Mathematica* under the function *FindIntegerNullVector* gives only one solution which is 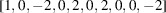. We notice that this vector is larger than all the vectors 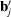 in columns 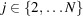 of the matrix 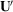.

The matrix 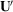 is interpreted crystallographically/geometrically as the unit cell attached to the hyperplane **p**. From an algebraic point of view, 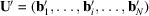 can equivalently be understood as the infinite set of solutions of the *N*-dimensional Bézout’s identity, where 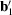 is a short solution of the equation 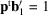, and the other vectors are short solutions of the integer relation 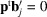, 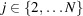. The set of solutions of Bézout’s identity is thus 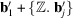 with 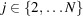, where {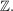} means all the linear combinations with integer coefficients. This 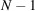-dimensional affine space represents all the solutions of Bézout’s identity made on the coordinates of **p**.

## 5. Conclusion   

The problem treated in the present paper called the ‘hyperplane unit-cell problem’ consists of finding, for any hyperplane **p** of *N* dimensions, one short vector 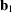 that is such that 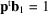 and 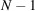 short integer vectors 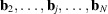 that are such that 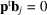. The short out-of-plane vector 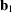 is the solution of Bezout’s identity with **p**, and the short in-plane vectors 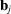, 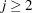, are solutions of the integer relation with **p**. These vectors constitute a unit cell attached to the hyperplane **p**. The algorithm to find a short solution to the *N*-dimensional Bézout’s identity is presented in Section 2. The algorithm to find a solution to a connected problem called the column-constrained unimodular matrix (CCUM) is detailed in Section 3. Both algorithms are then combined with the help of an oblique projection to determine a small unit cell attached to any hyperplane **p** (Section 4). The vectors 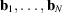 are short and can be further shortened by lattice reduction. We have shown in some examples that the solutions of the integer relation are even shorter than those determined by the PSLQ algorithm computed with *Mathematica*.

## Figures and Tables

**Table 1:** Equivalence of mathematic/crystallographic terms

*Mathematics*	*Crystallography*
Bézout’s identity: given an integer vector **p**, find an integer vector {\bf b}_{1} such that that {\bf p}^{\rm t}{\bf b}_{1} = 1	Given a plane **p**, find a lattice vector {\bf b}_{1} that points to a node of the first layer of **p**. The vector {\bf b}_{1} represents a translation between the layer *q* = 0 and *q* = 1
	
Integer relation: given an integer vector **p**, find N - 1 integer vectors {\bf b}_{j} such that that {\bf p}^{\rm t}{\bf b}_{j} = 0	Given a plane **p**, find N - 1 lattice vectors {\bf b}_{j} that lie in the layer *q* = 0 of the plane **p**
	
Set of solutions of Bézout’s identity {\bf b}_{1}+\{{\bb Z}.\ {\bf b}_{j}\} with j \in \{ 2,\ldots N\}	The lattice unit cell made of vectors {\bf{b}}_1,{\bf{b}}_2, \ldots, {\bf{b}}_N
	
Lattice reduction of the unit cell attached to **p**: find the vectors {\bf{b}}_1^\prime and {\bf{b}}_j^\prime as short as possible and such that {\bf{p}}^{\rm{t}}{\bf{b}}_1^\prime = 1 and {\bf{p}}^{\rm{t}}{\bf{b}}_j^\prime = 0	Lattice reduction of the unit cell attached to **p**: find a unit cell such that the vector pointing to the first layer and the in-plane vectors are as short as possible

## Appendix A Notations, Bézout’s identity and integer relations

We note  the *i*th coordinate of a vector . Sometimes, the notation  will be equivalently used. It should not be confused with  which is the *i*th vector in a set of vectors . The coordinates of a vector  are written in columns and those of a vector  are in rows. From a crystallographic point of view, column and row vectors belong to direct and reciprocal spaces, respectively. The matrix multiplication notation is adopted. It means that even a ‘simple’ scalar (inner) product  is written  where  means transpose of .

Bézout’s identity in 2D is an arithmetic theorem that states that for *a* and *b* which are integers with *d* for greatest common divisor, there exist integers *u* and *v* such that . More generally, the linear combinations of the form  are exactly the multiples of *d*. It can be generalized to any dimension *N* and written as follows. For any integer vector , calling *d* the greatest common divisor of the coordinates of , there exist integer vectors **u** such that . In the paper, we suppose that the coordinates of **p** are coprime, *i.e. d* = 1.

An integer relation between a real vector **x** exists if and only if there is an integer vector **u** such that . There are different algorithms to determine integer relations, such as the PSLQ (Ferguson *et al.*, 1999 ▸). Searching for an integer relation between a set of powers of *x* {1, *x*, *x*_2_,…, *x*_*n*_} permits one to determine whether a given real number *x* is likely to be algebraic. Integer relations are also searched between some mathematical constants such as e, π and ln(2) in order to establish new arithmetic conjectures. In the paper, only the cases where **x** is an integer vector (called **p**) are studied. (The equivalences between the mathematical and crystallographic terms used in the paper are given in Table 1 ▸.)
